# Comprehensive Genome-Wide Analysis of Thaumatin-Like Gene Family in Four Cotton Species and Functional Identification of *GhTLP19* Involved in Regulating Tolerance to *Verticillium dahlia* and Drought

**DOI:** 10.3389/fpls.2020.575015

**Published:** 2020-10-20

**Authors:** Zhanshuai Li, Xiaoyan Wang, Yupeng Cui, Kaikai Qiao, Longfu Zhu, Shuli Fan, Qifeng Ma

**Affiliations:** ^1^State Key Laboratory of Cotton Biology, Institute of Cotton Research of Chinese Academy of Agricultural Sciences, Anyang, China; ^2^National Key Laboratory of Crop Genetic Improvement, Huazhong Agricultural University, Wuhan, China; ^3^College of Biology and Food Engineering, Anyang Institute of Technology, Anyang, China

**Keywords:** cotton, thaumatin-like protein, expression patterns, *Verticillium dahliae*, drought, trichome

## Abstract

Thaumatin-like proteins (*TLP*s) present in the form of large multigene families play important roles in biotic stress and abiotic stress. However, there has been no systematic analysis of the *TLP*s in cotton. In this study, comprehensive identification and evolutionary analysis of *TLP*s in four species of cotton were conducted. In total, 50, 48, 91, and 90 homologous sequences were identified in *Gossypium raimondii*, *G. arboreum*, *G. barbadense*, and *G. hirsutum*, respectively. Gene structure, protein motifs, and gene expression were further investigated. Transcriptome and quantitative real-time PCR analysis indicated that *GhTLP*s participate in abiotic, biotic stress and cotton fiber development. *GhTLP19* on chromosome At05 was selected as a candidate gene for further study. When *GhTLP19* was silenced by virus-induced gene silencing (VIGS) in cotton, with the increase of malondialdehyde (MDA) content and the decrease of catalase (CAT) content, and as the increase of disease index (DI) and hyphae accumulation, the plants were more sensitive to drought and *Verticillium dahliae*. Furthermore, the *GhTLP19* overexpressing *Arabidopsis* transgenic lines exhibited higher proline content, thicker and longer trichomes and more tolerance to drought when compared to wild type. This study will provide a basis and reference for future research on their roles in stress tolerance and fiber development.

## Introduction

Terrestrial plants are constantly threatened by various pathogens (Li et al., 2016; Xin et al., 2016). Plants have evolved sophisticated defense mechanisms to effectively protect against pathogens (Gimenez-Ibanez and Solano, 2013; Kazan and Lyons, 2014). Recognition receptors for plasma membrane-localized interact with pathogen- and damage-associated molecular patterns or extracellular effectors, and the interaction of intracellular recognition receptors and effectors from the corresponding pathogens is the main strategy to fight against the pathogen (Li et al., 2017; Xu et al., 2017). Pathogen-related (PR) proteins, which are induced in response to pathogen invasion, are the main plant defense factors against pathogens (Dubey and Singh, 2018). PR5, also known as thaumatin-like protein, is a member of the 17 PR families (Zhao and Su, 2010). Most TLPs contain structures that are thought to their specific receptor binding for antifungal activity: the highly conserved G-x-[GF]-x-C-x-T-[GA]-D-C-x(1,2)-[GQ]-x(2,3)-C sequence, a REDDD (arginine, glutamic acid, and three aspartic acid residues) structure and sixteen or ten cysteine residues that form eight or five disulfide bonds to maintain the stability of protein structure (Hu and Reddy, 1997; Liu et al., 2010a, 2020). The antifungal activity of the TLPs might be related to their ability to penetrate the fungal membrane and cause perforation through enzyme activity (Liu et al., 2010a; Jiao et al., 2018). Related studies have also shown that the TLPs have strong glucanase activity to hydrolyze β-D-glucan, which is the major cell wall component of oomycetes (Latijnhouwers et al., 2003; Singh et al., 2017).

The *TLP*s play an important role in the defense system of plants against various biotic and abiotic stresses (Petre et al., 2011). The *ClTLP27* gene in watermelon could significantly inhibit the growth of various fungal pathogens, such as *Fusarium verticillioides* and *Didymella bryoniae* (Zhang et al., 2018). Overexpression of an *Ocimum basilicum PR5* family member (*ObTLP1*) in *Arabidopsis* not only increased the tolerance of transgenic plants to *Sclerotinia sclerotiorum* and *Botrytis cinerea* but also enhanced the resistance to methyl jasmonate (Misra et al., 2016). Overexpression of *AsPR5* from garlic could significantly improve the resistance of garlic and *Arabidopsis* to *B. cinerea* (Rout et al., 2016). Overexpression of *GbTLP1*, which is involved in cotton fiber secondary cell wall development, increased the resistance to *V. dahlia*, salt and drought in transgenic tobacco (Munis et al., 2010). A grape *VqTLP29* gene enhanced the stomatal closure immune response of transgenic lines in response to pathogen-related molecular patterns, increased the resistance of transgenic *Arabidopsis* to powdery mildew and *Pseudomonas syringae*, and might also play a role in the signaling pathways of jasmonic acid, salicylic acid and ethylene (Yan et al., 2017). *Di19* combined with the TACA (A/G) fragment of the *PR5* promoter to increase the expression level of *PR5* and thus enhance the drought resistance of *Arabidopsis* (Liu et al., 2013). In addition, *TLP* could improve plant resistance to abiotic stress and played a role in growth and development processes, such as floral organ formation (Neale et al., 1990) and seed germination (Seo et al., 2008).

Although there have been some relevant studies on the *TLP*s in plants, further research on these genes is still needed, especially in cotton. As an important industrial crop, cotton plays an important supporting role in the economy and textile industry. Among the cotton species, tetraploid *Gossypium hirsutum* and *G. barbadense* cultivated in agricultural production were formed by interspecific hybridization between A genomic variety *G. arboreum* and D genomic variety *G. raimondii* during the evolutionary process (Wendel et al., 2009). Cotton is widely cultivated around the world, with high production of *G. hirsutum*, accounting for 90% of total cotton produced in the world; *G. barbadense* is of high quality but low yield, accounting for only 5–8%; *G. arboreum* and *G. raimondii* are rarely cultivated (Zhang et al., 2015). *V. dahliae* is the main pathogen of cotton, and the disease resistance of most commercial varieties is poor, which seriously affects fiber quality and yield (Gong et al., 2017; Zhang et al., 2017; Ma et al., 2018, 2019b). However, little is known about the *TLP*s and their roles in tolerance to *V. dahliae* and fiber development in cotton. In this study, we identified and characterized the *TLP* gene family in sequenced cotton species. Then, we analyzed their gene structures, phylogenetic relationships, and expression patterns in various tissues and under *V. dahliae*, salt, PEG and cold stress; the *cis*-elements in the putative promoters; and transcription factor binding sites of the *TLP*s.

To further identify the *TLP*s function in cotton, *GhTLP19* was identified and cloned for study. We found that when *GhTLP19* was silenced by VIGS, the tolerance of plants to *V. dahliae* and drought decreased. Moreover, the transgenic *Arabidopsis* lines of overexpressing *GhTLP19* were more drought-tolerant and had thicker and longer trichomes. This study aimed to provide important and useful information for further studies on the interaction between cotton and fungal invasion, other abiotic stresses and developmental processes.

## Materials and Methods

### Member Identification and Sequence Analysis

The hidden Markov model (HMM) profile of the conserved thaumatin (THN) domain (PF00314) was downloaded from the Pfam database (Finn et al., 2016)^1^. The four cotton genomic data (*Gossypium arboreum*, JGI; *G. raimondii*, CRI; *G. hirsutum*, ZJU; *G. barbadense*, ZJU) were downloaded from the Cotton Functional Genomics Database (CottonFGD)^2^ (Zhu et al., 2017). The genomic data of other species were obtained from the Phytozome v12.1^3^. HMMER 3.0 and BLASTP were used to search the *TLP* genes in four genomes of cotton and other species. Then, we eliminated the redundant genes from the HMM and BLASTP searches. The remaining genes were further identified using the normal mode of the SMART database^4^ (Letunic et al., 2015). The basic information of the four cotton *TLP* genes was collected from CottonFGD (see footnote). The signal peptides and transmembrane (TM) domains were predicted with SignalP 5.0^5^ (Petersen et al., 2011), and TMHMM 2.0^6^ (Krogh et al., 2001), respectively. The subcellular localization of the *TLP* genes was predicted by using the CELLO v.2.5 server^7^.

### Gene Structure, Phylogenetic Tree, and Conserved Motif Analysis

The gene exon/intron structure information of the cotton *TLP* family was retrieved from CottonFGD, and the gene structures were graphically visualized using the GSDS2.0 web server^8^ (Hu et al., 2015). The conserved motifs of cotton *TLP* protein sequences were analyzed using the MEME program. All the identified *TLP* protein sequences from four *Gossypium* species, *Theobroma cacao*, *Arabidopsis*, and *Oryza sativa*, were aligned using ClustalX 2.0. The maximum likelihood (ML) and JTT method of MEGA 7.0 was used to construct the phylogenetic tree with the p-distance model and 1,000 bootstrap replications (Tamura et al., 2013).

### *Cis*-Elements and TFBSs Analysis

The 2,000 bp predicted upstream region of the initiation codon (ATG) of all *TLP*s was determined, and then, all the sequences were submitted to the PlantCARE database to identify the *cis*-elements (Lescot, 2002). The putative transcription factor binding sites (TFBSs) of the *GhTLP* gene promoter regions were predicted using the Binding Site Prediction tool in the PlantTFDB 5.0 server^9^, with a strict criterion: threshold *p* ≤ 1 × 10^–6^.

### Transcriptome Data and qRT-PCR Analysis

Raw RNA-seq data of *G. hirsutum* acc. TM-1 were downloaded from the NCBI Sequence Read Archive (PRJNA248163). TopHat (Kim et al., 2013) and Cufflinks (Trapnell et al., 2012) were used for mapping reads and analyzing gene expression levels, and the value of the gene expression levels was normalized by fragments per kilobase million (FPKM).

The cultivated cotton lines CCRI36 (upland cotton with weak resistance to *V. dahliae*) and Hai7124 (sea-island cotton with strong resistance to *V. dahliae*) were grown in a greenhouse free of *V. dahliae*. The root dip method was used to inoculate two true-leaf stage cotton seedlings with *V. dahliae* (V991) at 2 × 10^7^ spores per ml. The roots of seedlings were collected from each sample at 0, 6, and 12 h after inoculation. Control plants were treated in the same way with sterile water. Fiber samples were taken from normally growing CCRI36 and Hai7124 plants at 5, 10 and 15 days after flowering. All samples collected were immediately flash-frozen with liquid nitrogen and stored in a −80°C freezer for RNA isolation. The total RNA of all samples was isolated by the RNAprep Pure Plant kit (Tiangen, Beijing, China), and the RNA was reverse transcribed to cDNA by the PrimerScript 1st Strand cDNA synthesis kit (TaKaRa, Dalian, China). qRT-PCR analysis of the *TLP*s was conducted using the SYBR Premix Ex Taq Kit (TaKaRa) and the ABI 7500 real-time PCR System (Applied Biosystems, Foster City, CA, United States), and the data were normalized using cotton ubiquitin 7 (*UBQ7*) as an internal control. The relative expression levels of the *TLP*s were calculated by the 2^–ΔΔCt^ method (Livak and Schmittgen, 2001; Wang et al., 2017). Genesis software^10^ was used to draw the heat-map. The color represents *TLP* expression profiles: Log2 (expression levels). Three biological replicates were obtained using 40 plants grown at a uniform growth stage. For each biological replicate, three technical replicates of each qRT-PCR reaction were applied.

### Cloning of *GhTLP19* and Transformation of *Arabidopsis*

The complete ORF of *GhTLP19* was cloned from cDNA of mixed samples of CCRI36 tissues. The ORF of *GhTLP19* was cloned into *pBI121* vector containing 35S promoter to construct 35S:*GhTLP19*. The 35S:*GhTLP19* vector was transferred to *Arabidopsis* (Colombia-0; WT) by *Agrobacterium*-mediated floral dip method (Clough and Bent, 1998). The positive transgenic lines were screened on 1/2 MS medium containing 50 ng/μl kanamycin. The mutant *atpr5* (*At1g75040*;SALK_052587C) was purchased from AraShare^11^. *Arabidopsis* seeds were sterilized with commercially diluted (1:1, V/V) sodium hypochlorite and then rinsed several times with sterile water. Germination was carried out on sterile 1/2 MS medium. The seedlings were transferred to soil and grown at 22°C under long-day conditions (16 h light and 8 h dark) after 7 days. The trichome of *Arabidopsis* leaves (WT, *atpr5*, and 35S:*GhTLP19*) was observed and photographed in real-time using an OLYMPUS BX53 microscope, and the diameter and length of the trichome were measured in real-time using the measurement tool of cellSens Standard software.

### VIGS and Stresses Treatment

A 313bp fragment of *GhTLP19* ORF was amplified and cloned into the pCLCrVA vector. The vectors (CLCrV:*GhTLP19*, empty vector CLCrV:*00* and CLCrVB) were transformed into *A. tumefaciens* strain LBA4404. The OD_600_ of activated agrobacterium was adjusted to 1.5 with the infiltration buffer (10 mM MgCl_2_, 10 mM MES, and 200 μM acetosyringone). We mixed CLCrV:*GhTLP19* and CLCrVB, CLCrV:*00* and CLCrVB, CLCrV:*GhPDS* and CLCrVB in a 1:1 ratio, then agroinfiltrated into fully unfolded cotton cotyledons of CCRI36 (Hu et al., 2018). After a night of darkness, the plants were transferred to a greenhouse at 25°C with a 16 h light/8 h dark photoperiod. When CLCrV:*GhPDS* plants showed phenotype, CLCrV:*GhTLP19* and CLCrV:*00* plants were treated with drought and *V. dahliae*, respectively. VIGS plants were irrigated with 15% PEG6000 to simulate drought treatment. The transgenic *Arabidopsis* and WT plants were also irrigated with 15% PEG6000 to simulate the drought environment. The *V. dahliae* (V991) was grown on PDA medium for 6 days at 25°C. Some mycelia were selected and cultured in Czapek’s liquid medium at 150 rpm in a shaking at 25°C for about 5 days. Finally, VIGS plants were infected with *V. dahliae* spore suspension with concentration of 2 × 10^7^ by root dip method (Miao et al., 2019). The contents of MDA and CAT were determined using Malondialdehyde (MDA) Assay Kit and Micro Catalase (CAT) Assay Kit (Solarbio, Beijing, China), respectively. At least 30 strains of each treatment were investigated for disease index. Three independent biological replicates were included for each sample in the experiment.

## Results

### Member Identification, Sequence Analysis and Phylogenetic Tree of *TLP*s

We identified 90, 91, 48, and 50 full-length putative *TLP*s in *G. hirsutum*, *G. barbadense*, *G. arboretum*, and *G. raimondii*, respectively. Based on their locations on the chromosomes, the *TLP* family members of the four cotton species were renamed *GhTLP1* to *GhTLP90*, *GbTLP1* to *GbTLP91*, *GaTLP1* to *GaTLP48*, and *GrTLP1* to *GTLP50*. The subcellular localization results showed that most of the TLP proteins were mainly located in the extracellular space, just a few were located in the periplasmic and outer-membrane. Most TLPs contained an N-terminal signal peptide. The prediction results of the TM domains showed that half of the TLPs possessed one or two TM domains, while only GhTLP12 possessed four TM domains, and GhTLP55 and GbTLP53 possessed three TM domains. The molecular weight (MW), isoelectric point (pI) and grand average of hydropathicity (GRAVY) of the *TLP*s of the four cotton species were analyzed and are shown in Supplementary Table S1.

**FIGURE 1:**
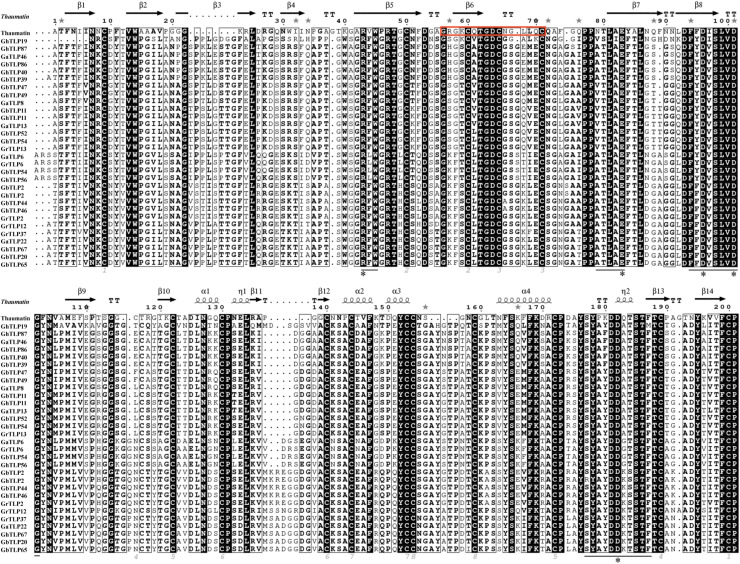
Thaumatin sequence alignment of partial *TLP*s in cotton. The *TLP* family signature in thaumatin is framed with a red border. The conserved residues are indicated by gray numbers. Conserved positions of five amino acids are labeled with a black asterisk. The black line shows the amino acids forming the bottom of the acidic cleft.

**FIGURE 2:**
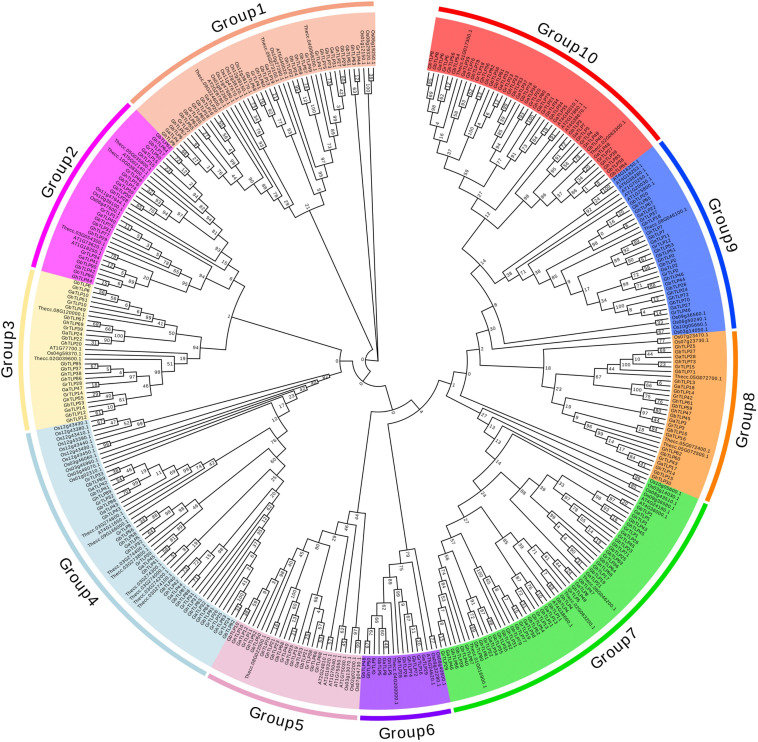
Phylogenetic tree of Thaumatin-like proteins. All predicted protein sequences from *G. raimondii*, *G. arboreum*, *G. hirsutum*, *G. barbadense*, *Arabidopsis*, *T. cacao*, and *O. sativa* were aligned with ClustalX 2.0, and the maximum likelihood (ML) and JTT method of MEGA 7.0 was used to construct the phylogenetic tree with the p-distance model and 1,000 bootstrap replications. Ten groups were indicated using different background colors.

Through multiple sequence analysis, we found that most TLP members contained conserved cysteine residues (Figure 1 and Supplementary Figure S1). Highly conserved REDDD amino acids and typical thaumatin family signature, which are essential for the antifungal and structural stability of the proteins under extreme environmental conditions (Fierens et al., 2009), also exist in most TLP family members. To study the evolutionary relationship of the *TLP*s, we collected *TLP* protein sequences from *G. raimondii*, *G. arboreum*, *G. hirsutum*, *G. barbadense*, *A. thaliana*, *T. cacao*, and *O. sativa* to construct a phylogenetic tree. On the basis of previous *AtTLP* and *CmTLP* family studies (Liu et al., 2010a, 2020), the phylogenetic tree was divided into 10 groups (Figure 2). All members of Group10 were derived from dicotyledonous plants, while the other nine groups included members of both dicotyledonous and monocotyledonous plants. The distribution of the cotton *TLP* members in all groups was relatively uneven. Group7 had 58 members, making it the largest group. Group6 is the smallest group, with only sixteen members.

To further elucidate the structural diversity of *TLP*s in cotton, we first analyzed the intron/exon structure and phylogenetics of the *GhTLP*s (Supplementary Figures S2A,B). The results showed that the number of introns in the *GhTLP*s was small, which is similar to results in *Solanum nigrum* and *Cucumis melo* (Jami et al., 2007; Liu et al., 2020). These individual genes with multiple exons contained other domains (such as PF11891 and PF00069) in addition to the THN domain. The conserved region of a protein determines its function. Therefore, the conserved motifs of *GhTLP*s were also predicted (Supplementary Figure S2C). A total of 10 highly conserved motifs were discovered, and most *GhTLP*s contained them. Each motif was located on 80% or more of the *GhTLP*s. Motif 5, 6, and 8, for example, existed on all *GhTLP*s. Members of the same group had nearly identical motifs, suggesting that they may be functionally identical or similar. We also analyzed the gene structure and motifs of all cotton *TLP*s (Supplementary Figure S3). The results showed that the *TLP*s were significantly conserved in cotton.

### Analysis of *Cis*-Acting Elements and TFBS

Transcription factors (TFs) are protein molecules that bind to specific sequences of a gene promoter (mainly *cis*-acting elements) to ensure specific temporal and spatial expression of the target gene at a specific intensity. To further elucidate the regulatory mechanism of *TLP* expression, we identified all *cis*-acting elements within 2000 bp upstream of the start codons of the *TLP*s (Supplementary Figures S4, S5 and Supplementary Table S2) and predicted the corresponding TFs of the *GhTLP*s (Supplementary Table S3). We found that the different *cis*-acting elements in the *TLP* gene promoters had the same proportion among the four cotton varieties (Supplementary Figure S4A), suggesting that the *TLP*s had similar expression regulatory patterns among these species. The statistical results showed that the sum of different *cis*-acting elements in tetraploid cotton (*G. hirsutum* and *G. barbadense*) was twice that in diploid cotton (*G. raimondii* and *G. arboreum*) (Supplementary Figure S4B), which supported the evolutionary theory that chromosome doubling resulted in tetraploid formation of the two diploid cotton species by hybridization. Since the number and category of *cis*-acting elements in the two tetraploid cotton species were basically the same and were twice as many as those in the two diploid cottons, we only performed the distribution map of the *cis*-acting elements in the *GhTLP* promoters (Supplementary Figure S5). Various plant hormone-related elements, such as ABRE (ABA), CGTAC-motif (MeJA), ERE (ethylene), TGA-element (auxin), and TATC-box (gibberellin), and stress-related elements, such as LTR (cold), MBS (drought), ARE (anaerobic), TC-rich (defense and stress), as-1, and WUN-motif, were identified. Among the plant hormone-related components, ERE components had the highest number and can respond to ethylene, disease and insect damage. There were also large numbers of SA elements, and the synthesis of SA can improve the resistance of cotton to *V. dahliae*. As an important component for the response to damage and pathogens, the W-box is necessary for the response of the *TLP* gene promoter to fungal elicitors, as well as many *GhTLP* promoters. These results suggested that *TLP*s might be involved in the resistance of cotton to pests and diseases. The binding sites of MYB and MYC-like TFs on various antistress gene promoters were abundant on the *TLP* promoters. There were also tissue-specific components, such as the RY-element, O2-site, and CCGTCC motif.

The TFBS results showed that a total of 308 TFs belonging to 40 families might combine with 90 promoter regions of *GhTLP*s (Supplementary Table S3). There are many plant-specific TF families, such as TCP, Dof, BBR-BPC, NAC, and B3. Some TF families, such as the MYB, ERF, and C2H2 families, are found not only in plants but also in animals, yeast and bacteria. These TFs interact with *cis*-acting elements on the promoter to regulate biological processes, including biotic/abiotic stress, plant growth and development. For example, ERF can bind to the *cis*-acting elements LTRE and DRE to respond to low temperature, drought and high salt stress. bZIP can interact with the *cis*-acting elements ABRE and as-1 in response to wounding, ABA, and MeJA. In addition, we analyzed the GO annotation information of the transcription factors of the *GhTLP*s (Supplementary Figure S6). The results showed that these TF families were mainly involved in biological processes and molecular functions, while fewer TF families were involved in cellular components. The main biological processes involving in these TFs were cellular process, metabolic process, biological regulation, and regulation of biological process. Among the molecular functions, binding and nucleic acid binding transcription factor activity are involved in the regulatory processes of these transcription factors. The number of TFs and *cis*-acting elements of the *TLP*s was very high; these elements might not only participate in cotton resistance to biotic stress (such as *V. dahliae*) and abiotic stress (drought, salt, and cold, etc.) but also might affect the growth and development process.

### Expression Patterns During Growth and Development

**FIGURE 3:**
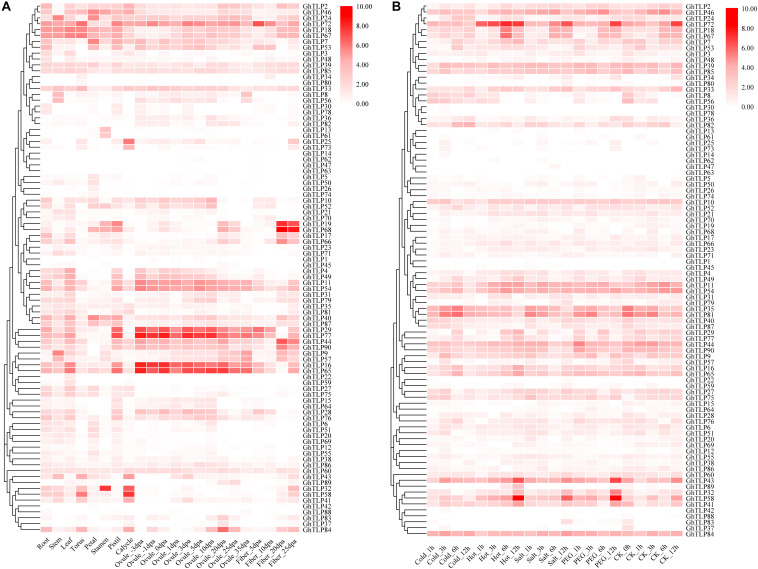
Expression profiles of the *GhTLP*s in different tissues **(A)** and in response to different stresses **(B)** of TM-1. The raw data for RNA-Seq of *G. hirsutum* acc. TM-1 was downloaded and used to analyze the expression patterns of *ChTLP* genes. The color bar represents the expression values in log2 of fragments per kilobase per million reads (FPKMs).

**FIGURE 4:**
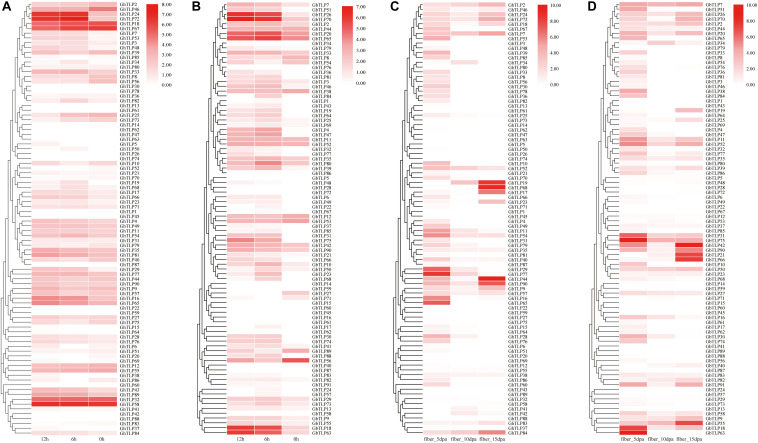
The expression patterns of the *TLP*s under *V. dahliae* treatment and during fiber development via qRT-PCR. **(A,B)** Show the expression patterns of the *TLP*s at 0, 6, and 12 h after CCRI36 and Hai7124 were inoculated with *V. dahliae*, respectively. **(C,D)** Show the expression profiles of the *TLP*s in 5 dpa, 10 dpa and 15 dpa fibers of CCRI36 and Hai7124 after flowering, respectively. The color represents *TLP* expression profiles: Log2 (expression levels). Three independent biological replicates and three technical replicates for each PCR reaction were included.

Analyzing gene expression patterns is a key method for exploring gene function. To explore the spatio-temporal expression patterns of *TLP* genes, the RNA-seq, and qRT-PCR data from different tissues and developmental stages were used to generate heat-maps. The expression pattern of each *TLP* gene was significantly different across different tissues and developmental stages (Figures 3A, 4C,D, Supplementary Figure S7 and Supplementary Table S4). Some genes were specifically expressed in the reproductive organs (Figure 3A). *GhTLP7*, *GhTLP40*, and *GhTLP46* exhibited preferential gene expression in the petals, *GhTLP25* and *GhTLP58* were specifically expressed in the calycles, and *GhTLP46* showed strong expression in the stamens. Four genes (*GhTLP29*, *GhTLP77*, *GhTLP16*, and *GhTLP65*) were highly expressed continuously and specifically during ovule development. *GhTLP84* was highly expressed only in ovules 20 days post-anthesis (dpa). These genes might be involved in the development of cotton reproductive organs. These results indicated that these five genes played a corresponding role in ovule development. Fiber is an important indicator of cotton yield and quality, and it is very important to analyze gene expression in fiber development. *GhTLP72* exhibited preferential gene expression in fiber_5 dpa and then gradually decreased, indicating that this gene might be involved in the fiber elongation stage of development. *GhTLP19*, *GhTLP68*, and *GhTLP44* showed the strongest gene expression in fiber at 20 and 25 dpa, suggesting that these genes played a role in the development of the fiber secondary cell wall.

*GbTLP1* has been reported to be involved in the development of fibers (Munis et al., 2010). To further confirm the role of *TLP*s in fiber elongation and secondary wall thickening, we collected fiber samples of upland and sea-island cotton 5, 10, and 15 days after flowering and then analyzed the *TLP* expression patterns by qRT-PCR (Figures 4C,D and Supplementary Tables S4, S6). In upland cotton (Figure 4C), *GhTLP19*, *GhTLP68*, *GhTLP17*, *GhTLP44*, *GhTLP90*, *GhTLP9*, and *GhTLP84* were specifically expressed at high levels in fiber_15 dpa. There were also many *GhTLP*s, such as *GhTLP65*, *GhTLP16*, *GhTLP29*, and *GhTLP77*, specifically and highly expressed during the fiber_5 dpa period. This finding was consistent with the results of public data transcripts (Figure 3A). In sea-island cotton (Figure 4D), genes such as *GbTLP55*, *GbTLP42*, *GbTLP21*, and *GbTLP66* were highly expressed during the fiber_15 dpa period, while the expression levels of *GbTLP63*, *GbTLP18*, *GbTLP75*, and *GbTLP31* were significantly upregulated during the fiber 5 dpa period. These results suggested that the *TLP*s might play a role in cotton fiber elongation and secondary wall development.

In addition, the expression levels of some genes (such as *GhTLP41*, *GhTLP43*, and *GhTLP89*) increased significantly during seed germination and in the roots but decreased gradually with cotyledon development (Supplementary Figure S7). There were also some genes (such as *GhTLP16* and *GhTLP65*) whose expression levels decreased with root development (Supplementary Figure S7). *GhTLP9* and *GhTLP24* were specifically expressed in the stems (Figure 3A). The results suggested that these genes played a role in cottonseed germination and seedling growth.

### Expression Patterns Under Biotic/Abiotic Stresses

It was found in plants that *TLP*s had a certain response to abiotic stress in many plants (Hiroyuki and Terauchi, 2008; Tachi et al., 2009; Goel et al., 2010; Munis et al., 2010; Misra et al., 2016). Therefore, we also used public RNA-seq and qRT-PCR data to analyze the expression patterns of the *GhTLP*s under abiotic and biotic stress (Figures 3B, 4A,B). Several genes showed increased expression levels under different stresses (Figure 3B). For example, *GhTLP35* and *GhTLP81* showed a tendency to increase and then decrease under salt, cold and PEG conditions. However, both *GhTLP58* and *GhTLP43* increased to the highest level after 12 h of salt and PEG treatment. The results showed that these genes could respond to different abiotic stresses to different degrees. Other *GhTLP*s also showed changes in expression levels under different abiotic stresses.

*TLP*s have been reported to play an important role in plant resistance to pathogen infection (Yan et al., 2017). To further explore the function of the *TLP*s in cotton resistance to *V. dahliae*, the expression patterns of the *TLP*s in CCRI35 and Hai7124 after *V. dahliae* inoculation were analyzed by qRT-PCR (Figures 4A,B and Supplementary Table S6). The results showed that many *TLP*s of CCRI36 and Hai7124 were rapidly upregulated under the induction of *V. dahlia*. In upland cotton (Figure 4A), *GhTLP24* and *GhTLP72* were most significantly upregulated. Some genes, such as *GhTLP84*, *GHTLP33*, *GhTLP19*, *GhTLP66*, *GhTLP16*, *GhTLP65*, *GhTLP43*, and *GhTLP89*, were also upregulated to some extent. There were also some genes whose expression was downregulated, such as *GhTLP46* and *GhTLP25*, and some whose expression levels were unchanged, such as *GhTLP32* and *GhTLP58*. In sea-island cotton (Figure 4B), most *TLP*s were upregulated under the induction of *V. dahlia*. For instance, the upregulation of *GbTLP18*, *GbTLP63*, *GbTLP26*, *GbTLP70*, *GbTLP20*, and *GbTLP65* was the most obvious. *GbTLP51*, *GbTLP4*, *GbTLP47*, *GbTLP75*, and *GbTLP74* were barely expressed at 0 h, but the expression levels were significantly upregulated after *V. dahlia* infection. These results indicated that many *TLP*s might participate in the resistance of cotton to *V. dahlia*.

### Ectopic Expression of *GhTLP19* Promotes the Development of Trichome and Resistance to Drought in *Arabidopsis*

**FIGURE 5:**
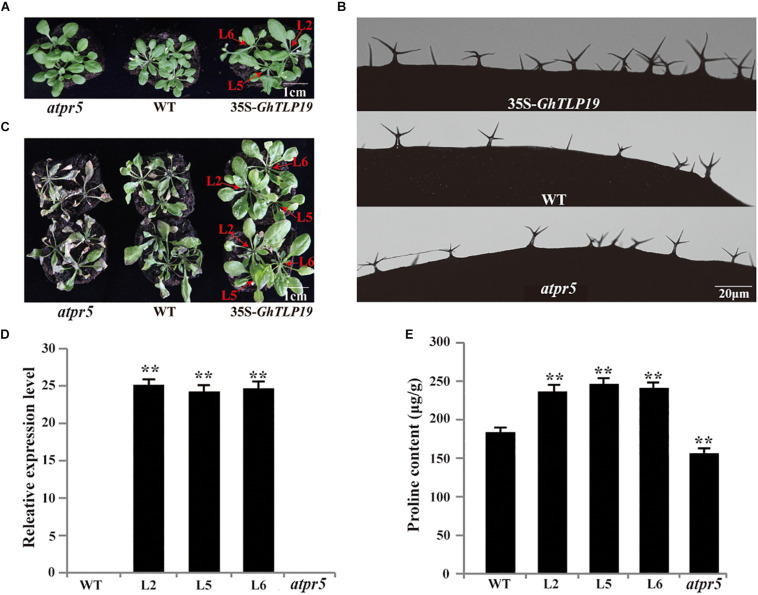
*GhTLP19* overexpression promotes the trichome development and confers tolerance to drought in *Arabidopsis*. **(A)** Normal growth of *Arabidopsis* transgenic lines overexpressing *GhTLP19*, WT and *atpr5*. **(B)** The morphology of trichome in *Arabidopsis* transgenic lines overexpressing *GhTLP19*, WT and *atpr5* under normal growth conditions. **(C)** *GhTLP19* overexpression improved tolerance to drought in *Arabidopsis*. The plants were treated with 15% PEG6000 for 10 days. **(D)** Identification of *Arabidopsis* transgenic lines overexpressing *GhTLP19*. **(E)** The proline contents of *Arabidopsis* transgenic lines overexpressing *GhTLP19*, WT and *atpr5* under drought stress. L2, L5, L6 represent CLCrV:*GhTLP19*-2, -5, -6 lines, respectively. The bars represent the means ± SEs from three independent experiments. **indicates statistical significance at the 0.01 probability level. Three biological replicates were used, one of which is represented. In each biological replicate, more than 20 plants were used.

Tissue expression patterns showed that *GhTLP19* was specifically highly expressed during fiber_15, 20, 25 dpa period. Due to the similar regulatory mechanism of cotton fiber and *Arabidopsis* trichome (Wang et al., 2004; Guan et al., 2014), *GhTLP19* was overexpressed in *Arabidopsis* to further explore its function in fiber development. Then, the relatively high expression of *GhTLP19* was selected from T3 generation transgenic lines to observe the trichome development (Figures 5A,B,D). At the same time, *Arabidopsis* T-DNA insertion mutant *atpr5* (*At1g75040*; SALK_052587C) of *GhTLP19* homologous gene was recruited to analyze the phenotype (Figures 5A–C). It was indicated that the leaf trichome of transgenic lines was thicker than that of WT, but the difference was not significant, and the mutant was obviously the thinnest. What is more obvious is that the branching length and diameter of leaf trichome of transgenic lines increased significantly compared with that of WT and mutants (Figure 5B and Table 1). The results showed that the ectopic expression of *GhTLP19* significantly promoted the development of *Arabidopsis* trichome. Cotton fiber and *Arabidopsis* trichome are both composed of single cells, and their development patterns are also very similar, so it is speculated that *GhTLP19* might participate in the development of cotton fiber.

**TABLE 1:** Compare the length and diameter of trichome.

Genotype	Length of trichome main stem/μm	Diameter of trichome main stem/μm	Length of trichome branch/μm	Diameter of trichome branch/μm	n
35S-*GhTLP19*	6.98 ± 2.15	2.56 ± 0.47	16.25 ± 4.13**	1.07 ± 0.08*	26
WT	5.34 ± 1.83	2.07 ± 0.31	10.58 ± 1.86	0.91 ± 0.06	27
Mutant	4.99 ± 0.95	1.01 ± 0.01*	5.98 ± 0.47**	0.65 ± 0.06**	23

Values represent the means and standard deviations of three biological replicates. * Indicates a significant difference from WT (p < 0.05). ** Indicates a significant difference from WT (p < 0.01). Data are presented as the mean ± SD.

The well-growing *GhTLP19* transgenic lines, WT and mutant were irrigated with 15% PEG6000 to observe their response to the drought conditions. After irrigating with 15% PEG6000 for 10 days, the results of the determination of proline content showed that the proline content of *GhTLP19* overexpressed lines was significantly higher than that of WT, while the proline content of mutant strains was significantly lower than that of WT (Figure 5E). The wilting phenomenon of WT leaves was obvious, and that of mutant leaves was the most serious, while the growth of *GhTLP19* overexpressed plants was relatively good (Figure 5C). Thus, overexpression of *GhTLP19* in *Arabidopsis* could improve the drought resistance of transgenic lines.

### Silencing *GhTLP19* Reduced Tolerance to Stress in Cotton

**FIGURE 6:**
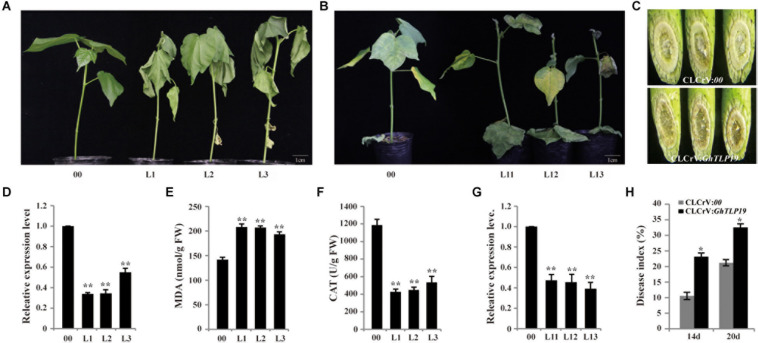
Silencing *GhTLP19* via VIGS increased sensitivity to drought and *V. dahliae* in cotton. **(A)** Phenotype of empty control and VIGS plants under 15% PEG6000 treatment. **(B)** Phenotype of empty control and VIGS plants under *V. dahliae* treatment. **(C)** V991 hyphal accumulation in WT and VIGS plants after inoculation. **(D)** The expression level of *GhTLP19* in empty control and VIGS plants to drought treat. **(E)** The MDA content of empty control and VIGS plants under drought stress. **(F)** The CAT content of empty control and VIGS plants under drought stress. **(G)** The expression level of *GhTLP19* in empty control and VIGS plants to *V. dahliae* treat. **(H)** Disease indices of CLCrV:*00* and CLCrV:*GhTLP19* plants were determined after inoculation with *V. dahliae*. L1, L2, L3, L11, L12, L13 represent CLCrV:*GhTLP19*-1, -2, -3, -11, -12, -13 lines, respectively. The bars represent the means ± SEs from three independent experiments. **and *indicate statistical significance at the 0.01 and 0.05 probability levels, respectively.

VIGS technology was used to reduce the expression level of *GhTLP19* to identify its role in cotton plant response to drought (Figure 6A) and *V. dahlia* (Figure 6B). The expression levels of *GhTLP19* in young leaves of control (CLCrV:*00*) and CLCrV:*GhTLP19* lines were measured after albino plaques appeared in the true leaves of plants inoculated with CLCrV:*PDS*-expressing agrobacteria, the results showed that the expression level of *GhTLP19* decreased significantly after VIGS (Figures 6D,G). The VIGS lines of CLCrV:*GhTLP19* (Line 1, 2, and 3) and CLCrV:*00* lines were treated with drought. Treated with 15% PEG6000 irrigation a week later, leaves of CLCrV:*GhTLP19* lines appeared obvious wilting phenotype, compared with the CLCrV:*00* (Figure 6A). Then we measured the contents of MDA and CAT in VIGS and control lines, and it was found that MDA content in VIGS lines was significantly higher than that in control lines (Figure 6E), while CAT content was significantly lower (Figure 6F). The results showed that silencing *GhTLP19* reduced cotton’s tolerance to drought.

qRT-PCR results showed that the expression level of *GhTLP19* was significantly up-regulated after CCRI36 was infected with *V. dahlia* (Figure 4A). VIGS technology was also used to silence the expression level of *GhTLP19* (Line 11, 12, and 13) to further identify its role in cotton response to *V. dahlia* (Figures 6B,C,G). After being infected with *V. dahlia*, CLCrV:*GhTLP19* lines showed obvious necrosis, yellowish and leaf shedding, while this phenomenon of the control lines was less severe (Figure 6B). Furthermore, the accumulation of mycelia in the stem of VIGS lines was significantly higher than that of the control lines (Figure 6C). The disease index (DI) of CLCrV:*GhTLP19* lines was significantly higher than that of control lines (Figure 6H). These data indicated that silencing *GhTLP19* could reduce cotton resistance to *V. dahlia*.

## Discussion

Our understanding of the genetic function of cotton, an economically important crop that can provide a large amount of industrial raw materials such as fiber, is limited (Ma et al., 2019a). As an important factor for plant resistance to biotic and abiotic stresses, *TLP*s may have a critical role in the regulation of the expression of many genes involved in the defense system (Wang et al., 2011; Singh et al., 2013; Zhang et al., 2018). However, there have been few reports on *TLP*s of cotton. In this study, we performed a systematic and comprehensive analysis of *TLP*s, and studied the function of *GhTLP19*.

### Characterization of the *TLP*s in Cotton

With the evolution of plants and the expansion of various gene families, plants have gradually increased their tolerance to various kinds of environmental stresses (Kohler et al., 2008). *TLP*s, found in many plants, are a gene family that resists both biotic and abiotic stress. Allotetraploid cotton species descended from interspecific hybridization events between their ancestors *G. raimondii* and *G. arboretum* (Wendel et al., 2009), and the number of *TLP*s in upland and island cotton should be equal to the sum of the *TLP*s in *G. raimondii* and *G. arboreum*. However, the actual number of *TLP*s in allotetraploids (*G. hirsutum* 90 and *G. barbadense* 91) was less than the sum of those in the two diploids (*G. raimondii* 50 and *G. arboreum* 48) (Supplementary Table S1), indicating that, the doubling of chromosomes and the rapid sequence arrangement of the genome would result in different degrees of gene loss in the process of polyploidization (Paterson et al., 2004). Sequence analysis results showed that most of the cotton TLPs contained highly conserved cysteine residues, typical thaumatin family signature, and REDDD amino acids, as well as signal peptides and transmembrane domains (Figure 1, Supplementary Figure S1, and Supplementary Table S1). The 16 cysteine residues form eight disulfide bonds that increase the protein stability and resistance to pH, proteases, and heat-induced denaturation (Ghosh and Chakrabarti, 2008; Fierens et al., 2009). The N-terminal signal peptides and transmembrane domains of the TLPs allow mature proteins to enter the secretory pathway and secrete extracellular proteins to participate in the defense response (Wang et al., 2011; Singh et al., 2013). The acid cleft domain composed of the conserved REDDD amino acids was thought to have antifungal activity after binding to specific receptors (Min et al., 2004). These sequential structural characteristics indicated that the *TLP*s in cotton might be involved in resistance to biotic stress, such as *V. dahliae*.

Evolutionary tree analysis showed that the *TLP*s from several species were divided into 10 groups, and the distribution of the *TLP*s was relatively dispersed in each subgroup (Figure 2), which was consistent with the distribution in melon and *Arabidopsis* (Liu et al., 2010a, 2020). The sequence characteristics of *TLP*s indicate that its family members should be paraphyletic and originate from 10 common ancestors before the differentiation of monocotyledons and dicotyledons (Shatters et al., 2006; Kohler et al., 2008; Liu et al., 2010b). The TLP motifs in cotton were highly conserved, basically containing 10 conservative motifs (Supplementary Figures S2, S3). In terms of gene structure, the *TLP*s have a relatively small number of introns, similar to those in melon (Liu et al., 2020) and black nightshade (Jami et al., 2007), except for a few genes containing other domains. These differences in the structure of individual genes might be related to the polyploidy of cotton chromosomes and the diversity of gene functions (Rogozin et al., 2003; Yang et al., 2008).

### Potential Regulatory Mechanisms and Functions of *TLP*s

As gene families expand and adapt to changes in the environment, members of a gene family might evolve new functions or increase/decrease the strength of old ones. By analyzing the expression patterns and regulatory relationships of family members, we further elucidated the potential functions of the *TLP* family in cotton. Our results suggested that the function of the *TLP*s in cotton might be not only related to resistance to biotic stress but also involved in abiotic stress, and plant growth and development.

For instance, studies have shown that *GbTLP1* was specifically expressed during the secondary wall development of cotton fiber (Munis et al., 2010), while we found that some genes, such as *GhTLP19*, *GhTLP68*, *GbTLP21*, and *GbTLP66* were specifically expressed in this period, and the expression levels were particularly high (fiber_15,20, and 25 dpa) (Figures 3A, 4C,D). In their promoter regions, all of these genes contained ERE regulatory elements (Supplementary Figures S4B, S5 and Supplementary Table S2) that could bind to ethylene transcription factors and regulate fiber development (Gou et al., 2007), and there were also many ERE-related TFs found in the TFBS analysis (Supplementary Table S3). *Cis*-elements (such as ABRE, TGA-element) and TFs (such as WRKY and Dof) can respond to ABA, IAA, and GA (Rushton et al., 2010) and participate in the growth and developmental processes of plants (Lijavetzky et al., 2003). The analysis of *cis*-element and TFBS revealed many specific elements that might be related to the development of seeds (RY-elements), endosperm (GCN4_motif) and TFs (Supplementary Figures S4B, S5 and Supplementary Tables S2, S3). Specific genes were also highly expressed in seeds (*GhTLP56* and *GhTLP82*) and roots (*GhTLP16* and *GhTLP65*) during seedling germination (Supplementary Figure S7). These results further suggested that the *TLP*s might be involved in the growth and development of cotton, especially reproductive and fiber development.

*TLP*s have also been found to respond to various biotic/abiotic stresses in other plants. Recently, study found that all *PR*-*5* genes might play a particular role in the defense system of soybean plants, especially leaves, against high salt stress (Tachi et al., 2009). *Arabidopsis* Di19, as a transcription factor, regulates the expression of *PR5* in response to drought stress (Liu et al., 2013). Overexpression of the osmotin gene increased the tolerance of transgenic tomato plants to salt and drought (Goel et al., 2010). In our expression profile of abiotic stress (Figure 3B), we found that *GhTLP35* and *GhTLP81* showed first increase then decrease trend under salt, cold and PEG conditions. Both *GhTLP58* and *GhTLP43* reached their highest expression levels after 12 h of heat, salt or PEG treatment (Figure 3B).

Moreover, study showed that ectopic expression of *ObTLP1* in *Arabidopsis* enhanced the tolerance to *S. sclerotiorum* and *B. cinerea* infection, as well as to dehydration and salt stress (Misra et al., 2016). Resistance to *V. dahliae* and *F. oxysporum* was significantly enhanced in transgenic plants overexpressing *GbTLP1*, and tolerance to salt and drought stress was also enhanced (Munis et al., 2010). As an important antimicrobial-related protein, *Rtlp1* can be induced by rice blast fungus, salicylic acid (SA) and methyl jasmonate (MeJA), and the W-box is an essential *cis*-acting element of the *Rtlp1* promoter in response to fungal elicitors (Hiroyuki and Terauchi, 2008; Miao et al., 2019). In our study, various elements that respond to SA (TCA-element), MeJA (CGTCA-motif, TGACG-motif) and the W-box were also found in the promoter regions of most *TLP*s and were necessary components for plants for induction of *TLP* expression by fungal elicitors. We also found that most *TLP* gene promoter regions in cotton contain *cis*-acting elements that respond to low temperature (LTR) and drought (MYC, DRE). In the TFBS analysis results (Supplementary Table S3 and Supplementary Figure S6), various transcription factors interacting with *TLP*s were related to plant resistance to biotic/abiotic stress. For example, the AP2 transcription factor could not only withstand abiotic stress such as drought and salt but also interacted with pathogen-related protein promoters to resist pathogen invasion (Park et al., 2001; Mizoi et al., 2012). ERF is also an important transcription factor in the response to important ethylene signals and pathogen invasion in plants (Licausi et al., 2010). The bZIP and C2H2 zinc finger protein transcription factors play a key role in the transcriptional regulation of plants in response to extreme temperature, salinity, drought and oxidative stress (Agarwal et al., 2019; Joo et al., 2019; Wang et al., 2019). The analysis results of these regulatory mechanisms indicate that *TLP*s might be involved in the process of cotton environmental stress.

Our qRT-PCR results showed that many *TLP*s (such as *GhTLP24*, *GhTLP72*, *GbTLP20*, and *GbTLP65*) were significantly upregulated in upland and sea-island cotton after *V. dahliae* infection (Figures 4A,B). Our experimental data showed that *Arabidopsis* with overexpression of *GhTLP19* was more drought-resistant, and cotton with silencing of *GhTLP19* gene was more sensitive to drought and *V. dahliae*. The above results indicated that the *TLP*s should participate in biotic/abiotic stress processes in cotton.

### Function of *GhTLP19* in *Arabidopsis* and Cotton

The development mechanism of cotton fiber and leaf trichome of *Arabidopsis* share many similarities (Lee et al., 2007). MiR828 and miR858 regulate the function of *MYB2* in *Arabidopsis* and cotton, respectively, to affect the development of trichome and fiber (Guan et al., 2014). Overexpression of *GhCLASP2* in *Arabidopsis* increased the number of trichome branches and restored the trichome phenotype of *clasp-1* mutant (Zhu et al., 2018). In our study, by overexpressing *GhTLP19* in *Arabidopsis*, the branches of the trichome are significantly longer than those of the WT. At the same time, the diameter of the trichome of the overexpressed *GhTLP19* lines was thicker than that of WT (Figure 5B and Table 1). We also found that the leaves of mutant and overexpressed lines were larger than those of wild-type. These results suggested that *GhTLP19* may play a role in the development of cotton fiber.

As previously mentioned, a large number of studies have shown that *PR* proteins play an important role in plant environmental stress (Munis et al., 2010; Misra et al., 2016). As a member of *PR* proteins, *GhTLP19* should also have certain resistance to environmental stress. In the current study, the overexpression lines and WT were irrigated with 15% PEG6000, and the results showed that the tolerance of the overexpressed lines to drought stress was significantly higher than that of WT, and the content of proline was also significantly increased (Figures 5C,E). Due to factors such as long time and low efficiency of cotton transgene, we chose short experimental period, simple operation method, low cost and high-efficiency VIGS technology to silence *GhTLP19* to study its functions (Gu et al., 2018). Thus we silenced *GhTLP19* in cotton with VIGS strategy and irrigated them with 15% PEG6000. The results showed that the silenced plants showed significant wilting than the control plants, and the content changes of MDA and CAT also reached a significant difference (Figures 6A,D,E,F). In order to identify the role of *GhTLP19* in the corresponding *V. dahliae* invasion of cotton, 2 × 10^7^ *V. dahliae* were used to infect VIGS and control plants (Figures 6B,G,H). The results showed that the resistance of VIGS plants to *V. dahliae* was weaker than that of control plants, the disease index and mycelia accumulation in the stems of VIGS plants were significantly higher than that of the control plants. This research is consistent with the research results of *GbTLP1* (Munis et al., 2010). Therefore, *GhTLP19* was also resistant to drought and *V. dahliae*. Further, *GhTLP19* co-expression network analysis found that a total of 26 genes were co-expressed with *GhTLP19* (Supplementary Figure S8). Through functional annotation (Supplementary Table S6), we found that the gene *Gh_A07G1075* (*CIPK1*)encoding serine-threonine protein kinase may improve the response of plants to environmental stress such as drought, high salt, wounding and abscisic acid (Pan et al., 2018). *Gh_D12G1855* and *Gh_A12G1694* (*CEP1*) may be involved in controlling late epidermal cell death, limiting growth, and increasing resistance to pathogen (Höwing et al., 2014). These genes may be co-expressed with *GhTLP19* and participate in the response to environmental stress (Figures 5, 6).

## Conclusion

In this study, we identified the *TLP*s in four different cotton varieties. Segmental and tandem duplication were the main mechanisms of gene expansion in the *TLP*s during evolution. The structural characteristics, evolutionary relationships, and conserved domains of these genes were also analyzed. In addition, we predicted and analyzed the potential molecular regulatory mechanisms and functions of the *TLP*s, as well as their expression patterns after the cotton species were infected with *V. dahliae*, during the fiber development period and in environmental conditions. The overexpression of *GhTLP19* in *Arabidopsis* and the VIGS experiment in cotton showed that *GhTLP19* could enhance plant resistance to drought and *V. dahliae*, and promote the development of *Arabidopsis* trichome and likely cotton fiber. These genes might be precisely regulated by transcription factors and various plant hormone signals in the external environment. Their functions are diverse; they not only respond to various biotic/abiotic stresses and hormone signaling pathways but also participate in some growth and development processes, such as fiber development.

## Data Availability Statement

The datasets presented in this study can be found in online repositories. The names of the repository/repositories and accession number(s) can be found in the article/ Supplementary Material.

## Author Contributions

QM and SF conceived and designed the experiments. KQ and XW prepared the figures and tables. LZ and YC analyzed and interpreted the data. ZL prepared the manuscript and participated in all experiments. KQ constructed the vector. LZ and QM treated and adjusted of traits. LZ and XW revised the manuscript. All authors have read and agreed to the published version of the manuscript.

## Conflict of Interest

The authors declare that the research was conducted in the absence of any commercial or financial relationships that could be construed as a potential conflict of interest.

## Footnotes

1 http://pfam.xfam.org

2 https://cottonfgd.org/

3 http://www.phytozome.net

4 http://smart.embl-heidelberg.de/

5 http://www.cbs.dtu.dk/services/SignalP/

6 http://www.cbs.dtu.dk/services/TMHMM/

7 http://cello.life.nctu.edu.tw

8 http://www.bioinfogenome.net/piece/

9 http://planttfdb.cbi.pku.edu.cn/

10 http://www.gsoft.com.au/

11 http://www.arashare.cn/

## Supplementary Material

Supplementary Figure 1 Thaumatin sequence alignment of all the *TLP* genes in cotton. The *TLP* family signature in thaumatin is framed with a black border. The conserved residues are indicated by cyan numbers. Conserved positions of five amino acids are labeled with a red asterisk. The black line shows the amino acids forming the bottom of the acidic cleft.

Supplementary Figure 2 Phylogenetic relationships, gene structure and motif analysis of the *GhTLP*s. **(A)** Phylogenetic relationships of the *GhTLP*s. **(B)** Gene structures of the *GhTLP*s. **(C)** The conserved motifs of the *GhTLP*s.

Supplementary Figure 3 Phylogenetic relationships, gene structures and motif analysis of the cotton *TLP*s. **(A)** Phylogenetic relationships and gene structures of the cotton *TLP*s. **(B)** The conserved motifs of the *TLP*s.

Supplementary Figure 4 Distribution characteristics of *cis*-acting elements in the promoter regions of *TLP*s. **(A)** The proportion of each *cis*-acting element in four different cotton species. **(B)** The number of *cis*-acting elements in each of the four cotton species.

Supplementary Figure 5 Distribution characteristics of the *cis*-acting elements in the promoter regions of the *GhTLP*s. Different *cis*-acting elements are represented by different color boxes.

Supplementary Figure 6 Gene Ontology (GO) annotation of the TFs binding to the *GhTLP* genes’ putative promoter regions based on their cellular component, molecular function, and biological process.

Supplementary Figure 7 Expression profile of the *GhTLP*s at the seed germination and seedling stages. The tissues are shown at the opening, genes are shown on the periphery, and the phylogenetic relationships are shown in the inner region. The color represents TLP expression profiles: Log2 (FPKM).

Supplementary Figure 8 Co-expression network of *GhTLP19*. Nodes representing individual genes and edges indicate significant co-expression between genes. The pink lines represent protein’s own interaction and positive co-expression relationship with the target protein. The blue lines represent protein’s own interaction and negative co-expression relationship with the target protein. The orange line represents protein own interaction and protein-protein relationship with the target protein. Prediction and analysis of co-expression networks, as well as gene function annotation, were performed on ccNET (http://structuralbiology.cau.edu.cn/gossypium/).

Supplementary Table 1 Details of the *TLP*s in four *Gossypium* species.

Supplementary Table 2 Distribution of the major *cis*-elements in the promoter regions of the *TLP*s. The locations of these *cis*-elements were confirmed using the PlantCARE database.

Supplementary Table 3 List of the identified TFBSs in the putative promoter regions of the *GhTLP* gene family using PlantTFDB 4.0.

Supplementary Table 4 The expression levels of the *TLP*s under *V. dahliae* treatment and during fiber development via qRT-PCR. The expression levels of each target gene were calculated by the cycle threshold (CT)^2–ΔΔCt^ method.

Supplementary Table 5 Primers for qRT-PCR in this study.

Supplementary Table 6 The annotation of *GhTLP19* co-expression genes.
