# Supplementary figures and images for: Comprehensive Genome-Wide Analysis of Thaumatin-Like Gene Family in Four Cotton Species and Functional Identification of GhTLP19 Involved in Regulating Tolerance to Verticillium dahlia and Drought
Source: Front Plant Sci. 2020 Oct 20;11:575015. doi: 10.3389/fpls.2020.575015 (PMC7606878; doi:10.3389/fpls.2020.575015)

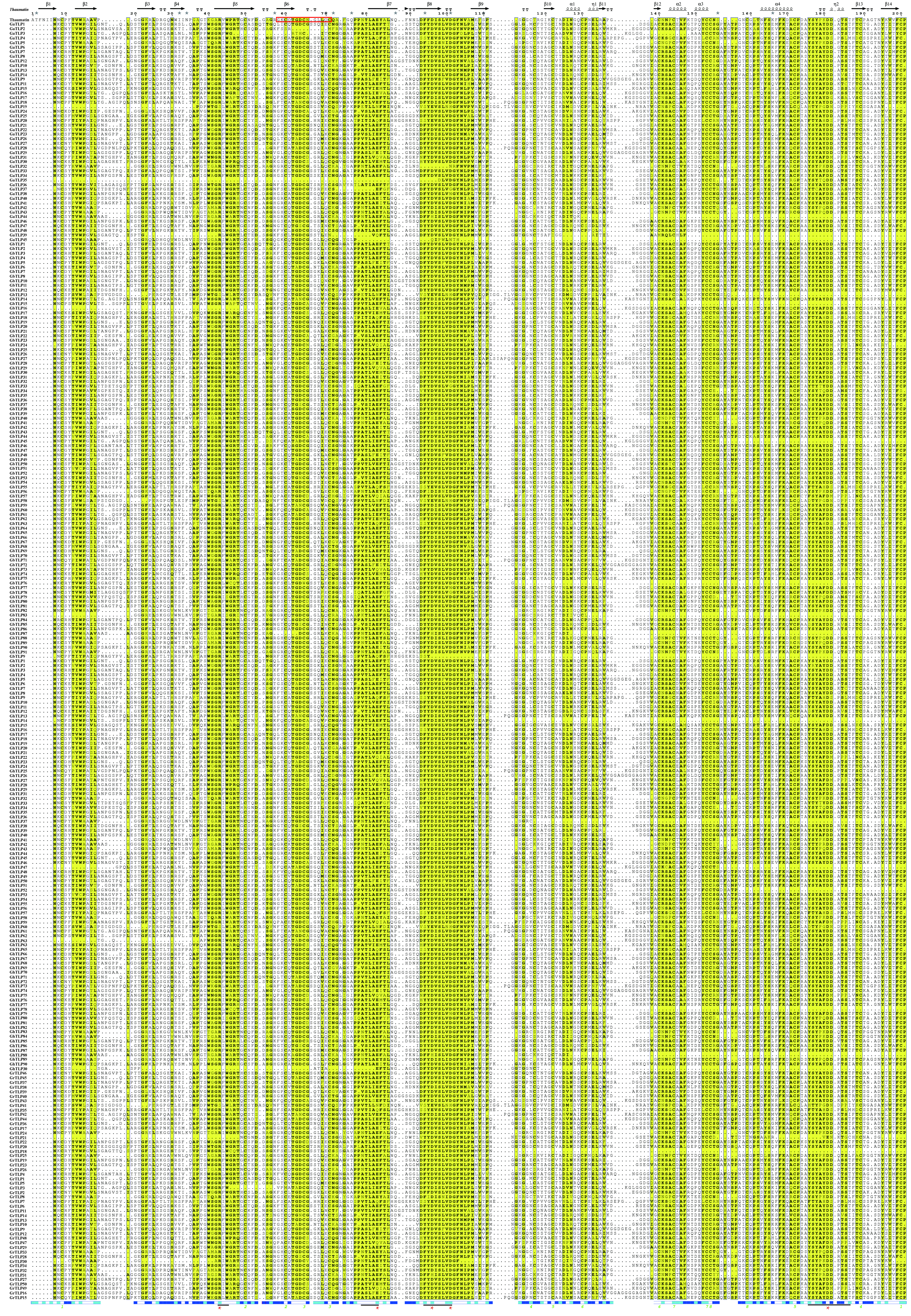

Supplement: Supplementary Figure 1 — Thaumatin sequence alignment of all the TLP genes in cotton. The TLP family signature in thaumatin is framed with a black border. The conserved residues are indicated by cyan numbers. Conserved positions of five amino acids are labeled with a red asterisk. The black line shows the amino acids forming the bottom of the acidic cleft. [file Image_1.TIF]

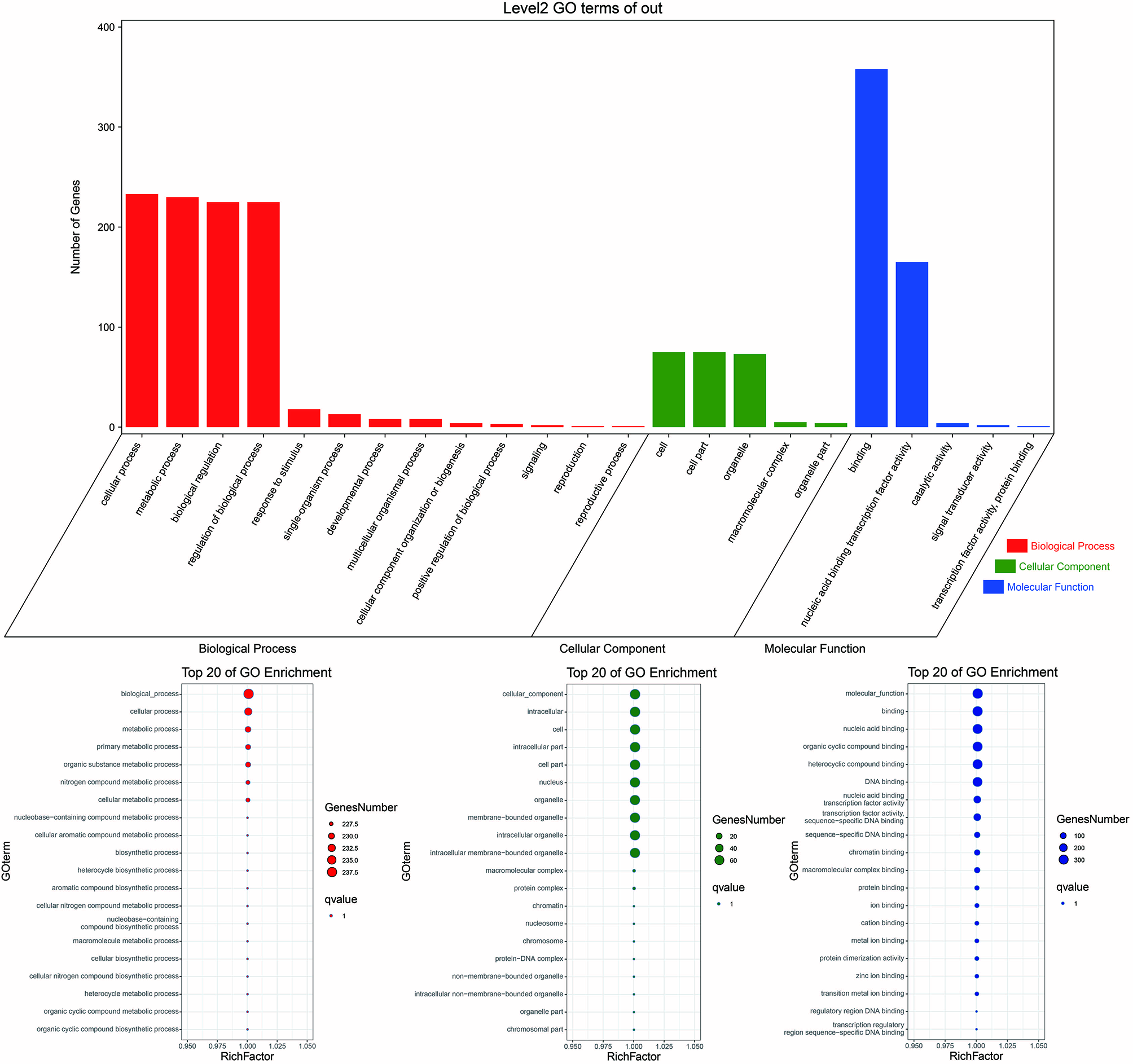

Supplement: Supplementary Figure 6 — Gene Ontology (GO) annotation of the TFs binding to the GhTLP genes’ putative promoter regions based on their cellular component, molecular function, and biological process. [file Image_6.TIF]

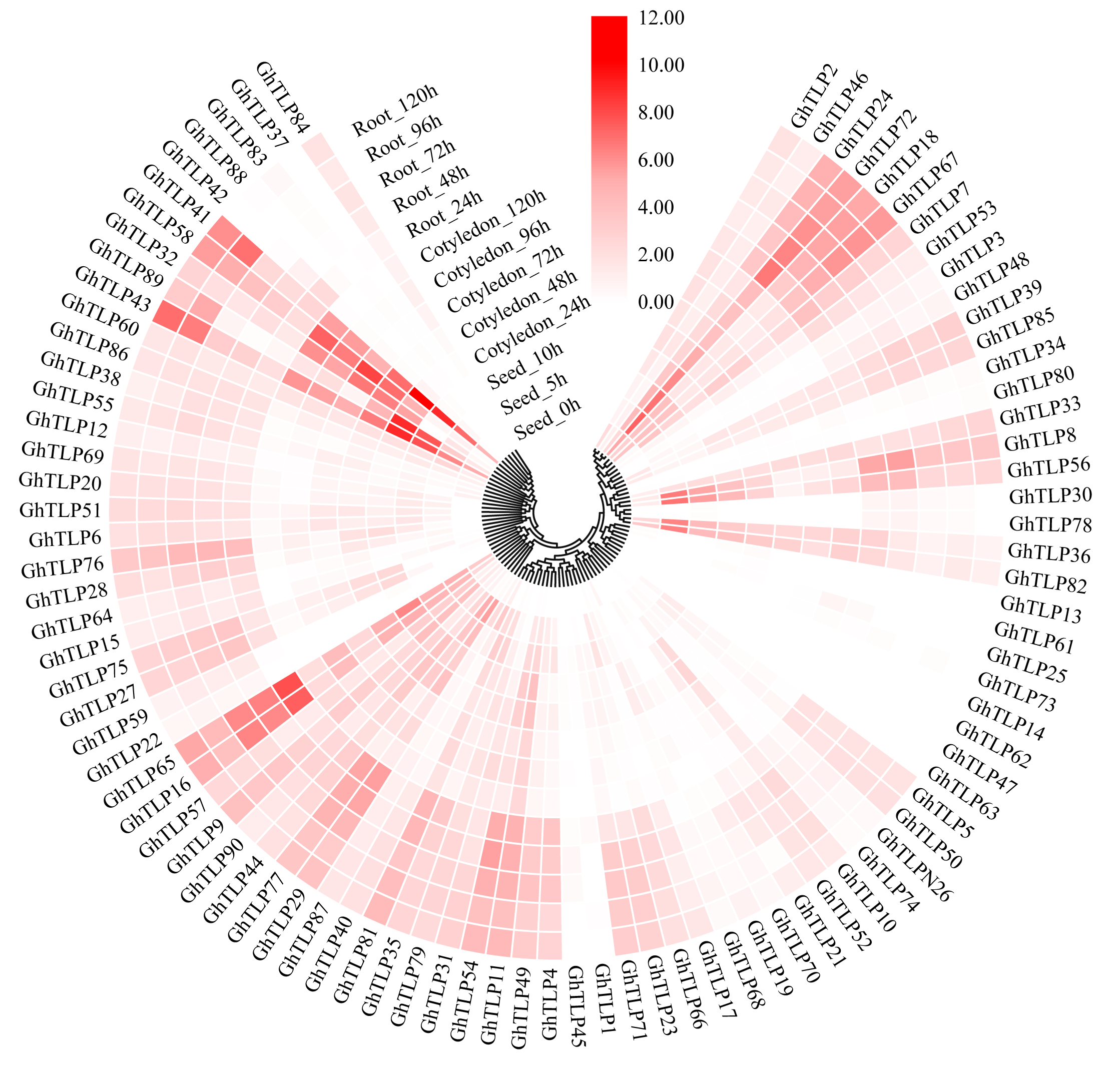

Supplement: Supplementary Figure 7 — Expression profile of the GhTLPs at the seed germination and seedling stages. The tissues are shown at the opening, genes are shown on the periphery, and the phylogenetic relationships are shown in the inner region. The color represents TLP expression profiles: Log2 (FPKM). [file Image_7.TIF]

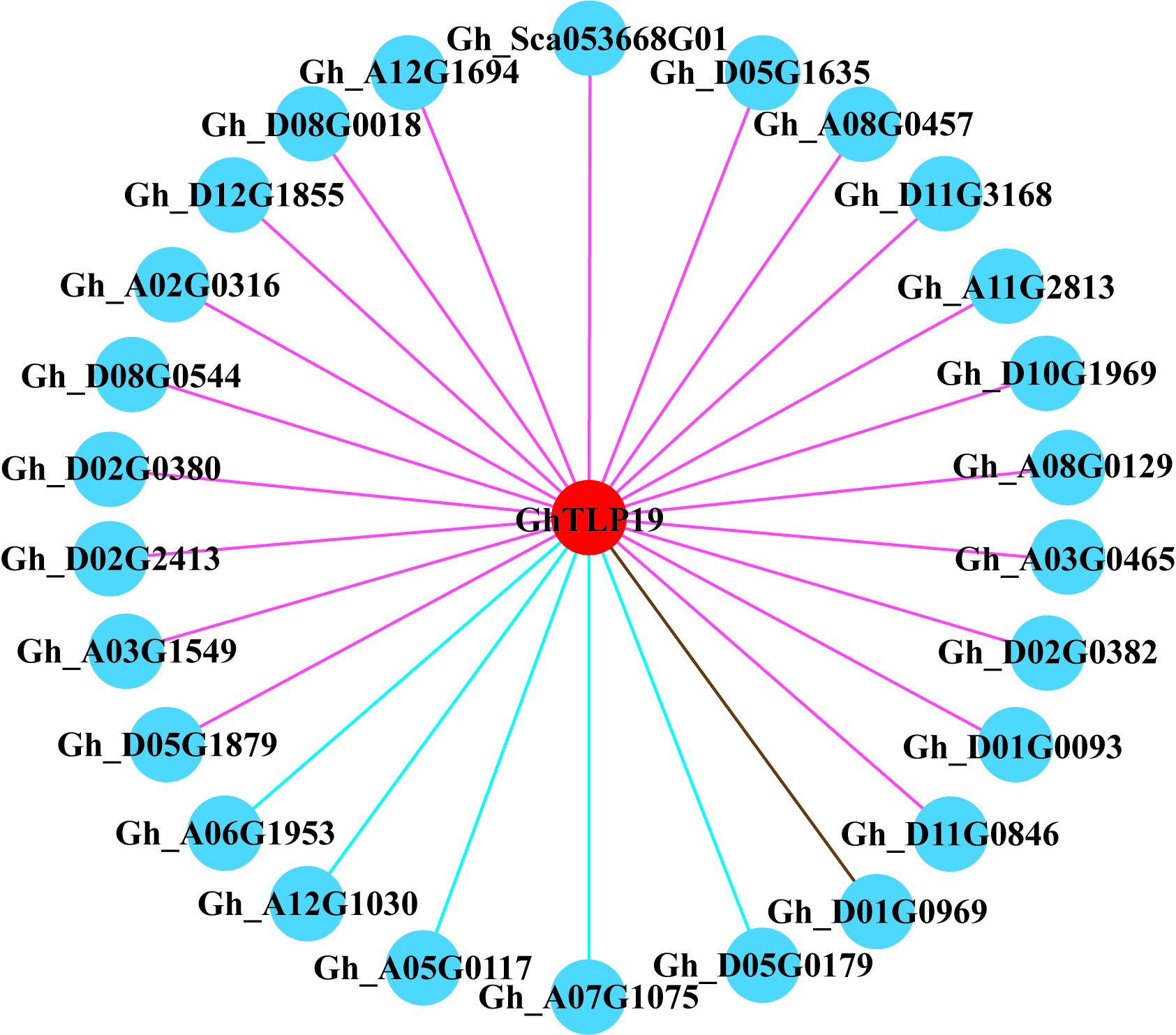

Supplement: Supplementary Figure 8 — Co-expression network of GhTLP19. Nodes representing individual genes and edges indicate significant co-expression between genes. The pink lines represent protein’s own interaction and positive co-expression relationship with the target protein. The blue lines represent protein’s own interaction and negative co-expression relationship with the target protein. The orange line represents protein own interaction and protein-protein relationship with the target protein. Prediction and analysis of co-expression networks, as well as gene function annotation, were performed on ccNET (http://structuralbiology.cau.edu.cn/gossypium/). [file Image_8.TIF]
